# Prevalence and its associated factors of depressive symptoms among Chinese college students during the COVID-19 pandemic

**DOI:** 10.1186/s12888-021-03066-9

**Published:** 2021-01-29

**Authors:** Mingli Yu, Fangqiong Tian, Qi Cui, Hui Wu

**Affiliations:** Department of Social Medicine, School of Public Health, China Medical University, Shenyang, 110122 China

**Keywords:** COVID-19, College students, Depressive symptoms, Online cross-sectional study

## Abstract

**Background:**

The COVID-19 pandemic has caused a mental health crisis around the world. The psychological health of college students also faces great challenges. This study aimed to investigate the prevalence and the related factors of depressive symptoms among Chinese college students.

**Methods:**

This online cross-sectional survey was conducted via Wenjuanxing platform from March 3–15, 2020 and received 1681 effective questionnaires. Each questionnaire contains the Center for Epidemiologic Studies Depression scale, the Multi-Dimensional Scale of Perceived Social Support, the Herth Hope Index, and the self-designed items. Multivariable logistic regression was conducted to determine the significantly associated factors of depressive symptoms.

**Results:**

The prevalence of depressive symptoms among college students was 56.8%. Sleep problems (OR 2.678, 95%CI 2.094–3.424), family members’ going out (OR 1.775, 95%CI 1.089–2.894), perceived more stress for online education (OR 1.642, 95%CI 1.191–2.263), fear of COVID-19 (OR 1.450, 95%CI 1.121–1.876), influence on social interaction (OR 1.354, 95%CI 1.053–1.741) and higher grade (OR 1.378, 95%CI 1.046–1.816) were considered as risk factors of depressive symptoms. Perceived social support (OR 0.354, 95%CI 0.259–0.484), hope (OR 0.052, 95%CI 0.034–0.080), female (OR:0.557, 95%CI 0.427–0.725) and higher monthly disposable income (OR 0.666, 95%CI 0.447–0.993) were identified as protective factors against depressive symptoms.

**Conclusions:**

There was a high prevalence of depressive symptoms among Chinese college students during the COVID-19 pandemic. It is important to find ways to alleviate the pressure and fear of college students, to provide them with more social support, and to help them adapt to the changes in learning style and lifestyle.

**Supplementary Information:**

The online version contains supplementary material available at 10.1186/s12888-021-03066-9.

## Background

As the greatest challenge humankind have faced since the Second World War, the COVID-19 pandemic has a huge impact on society, economy, health, environment and other aspects globally. As of September 14, 2020, the World Health Organization (WHO) have reported the 28 million confirmed cases globally and 917,417 cumulative deaths [1]. Apart from the reported physical damages, the COVID-19 pandemic has caused a mental health crisis around the world [2]. Some experts have pointed out that the psychological health of the public had been generally threatened [3].

Previous studies have shown that college students are more likely to have mental health problems [4]. The prevalence of depressive symptoms among college students is higher than that of general population or non-college students [5, 6]. Systematic reviews have indicated the prevalence of depressive symptoms among college students is about 33% [7], and the overall prevalence in Chinese college students is 23.8% [5]. Experts predict that the depressive problems of college students will become more serious [8]. For college students, depressive symptoms may lead to a decline in academic performance. Compared with healthy ones, college students with depressive symptoms on average are less likely to participate in organized activities, which may have a negative impact on their status in the social networks and even lead to an increase in suicidal tendency [9]. Therefore, the depressive symptoms of college students during the COVID-19 pandemic should be given more attention.

A generally accepted view is that college students’ depressive symptoms are the result of a combination of genetic, biological, psychological, interpersonal, and environmental factors [10]. Fear of the pandemic, home quarantine and the misinformation spread on the Internet are expected to aggravate psychological problems of college students [11]. What’s more, due to the delays in schools opening, Chinese college students have to continue their studies through online courses. For those with poor adaptability, the change of teaching methods may have a negative impact on their mental state.

With the development of positive psychology, scholars pay more attention to the positive psychological factors [12]. Some experts have already appealed for more researches from the perspective of positive psychology to deal with the mental health crisis caused by the COVID-19 pandemic [13]. Perceived social support (PSS) is one of the most important psychological resources to cope with challenging life events [14]. Social support has been reported to have a protective effect on college students’ mental health [15]. Furthermore, the inadequate social support may increase the risk of depressive symptoms among college students [16]. Moreover, as an internal psychological resource, hope is closely related to an individual’s goals and expectations for the future and can have a positive impact on one’s behavior and attitude [17]. Studies have shown that hope can moderate the relationship between negative life events and depressive symptoms [18]. When faced with life challenges, people with higher levels of hope are less likely to develop depressive symptoms. During home quarantine, the effects of PSS and hope on mental health of college students should not be ignored.

To sum up, this study was designed to investigate the prevalence of depressive symptoms among college students, and to explore its association with COVID-19-related perception and behavior, perception of online education, PSS and hope. In addition, we hope to provide a theoretical basis for the psychological interventions.

## Methods

### Ethics statement

This study was approved by the Committee on Human Experimentation of China Medical University (YDJK2020022). The study process was in accordance with the ethical standards. The purpose and process of this study were shown on the first page of the electronic questionnaire. This study gained informed consent from all the participants. Each participant in this survey need to provide his/her name, student number, school name, major and so on. The data obtained was kept confidential and anonymous to protect their privacy.

### Study design and sampling

This online cross-sectional study was conducted from March 3–15, 2020. Chinese college students were invited to participate in this web-based survey. By scanning QR code on Wechat, the participants entered the Wenjuanxing platform to complete electronic questionnaires. Finally, our electronic questionnaire was clicked 1775 times. After removing some incomplete questionnaires, 1681 Chinese college students from 33 provinces and autonomous regions became our research subjects. The response rate was 94.7%, eventually.

### Study variables and outcomes

In addition to depressive symptoms, PSS and hope, the self-designed items were used to describe the participants’ demographic characteristics, COVID-19-related perception and behavior, perception of online education (Additional file 1). Five demographic variables included gender, major, grade, region, and monthly disposable income. Major was divided into “medical related majors” and “others”. Grade was categorized into “1/2″ and “3/4/5″. Region included “rural” and “urban”. Monthly disposable income (yuan) was divided into “≤1000″, “1001–2000″ and “> 2000″.

COVID-19-related perception contains four variables, including information sources, fear of COVID-19, affected by global pandemic and influence on social interaction. COVID-19-related behavior consists of taking preventive medicine, sleep problems and going out. Perception of online education contains two variables: perceived more stress and overall satisfaction with online education.

### Data collection tools

The Center for Epidemiologic Studies Depression scale (CES-D) was used to evaluate depressive symptoms [19]. This scale contains 20 items to describe the frequency of the participants’ feelings in the past week. Each item has four options from 0 (rarely, less than 1 day) to 3 (most or all of the time, 5–7 days). The total score is from 0 to 60, with a higher score presenting more serious depressive symptoms. Individuals with a total score of 16 or more were considered to have depressive symptoms. CES-D has been demonstrated to have satisfactory validity and reliability for Chinese college students [20, 21]. In our study, the Cronbach’s alpha coefficient was 0.948.

To evaluate PSS, we adopted the Multi-Dimensional Scale of Perceived Social Support (MSPSS) [22]. This scale consists of 12 items, and each item scores from 1 (strongly disagree) to 7 (strongly agree). Higher total score means a higher level of PSS. The total score between 12 and 36 was defined as low support state, 37–60 as moderate support state, and 61–84 as high support state [23]. In this study, less than 2% (30) of the participants had a low level of PSS. Therefore, we combined college students in low support state and moderate support state into one group. Finally, the level of PSS was divided into two groups: “moderate” (summed score ≤ 60) and “high” (summed score ≥ 61). The Chinese version of MSPSS has been verified to have good reliability and validity [24, 25]. In this study, the Cronbach’s alpha coefficient was 0.960.

The Herth Hope Index (HHI) was chosen to evaluate the level of hope [26]. HHI contains 12 items with each item scoring from 1 (very disagree) to 4 (very agree). The total score is between 12 and 48. A total score from 12 to 23 indicates a low level of hope, 24 to 35 as a moderate level, and 36 to 48 as a high level [27]. Because less than 1% (12) of college students were at a low hope level in this study, we combined college students with a low hope level and a moderate hope level into one group. Eventually, the level of hope was categorized as “moderate” (total score ≤ 35) and “high” (total score ≥ 36). The HHI has been widely used at home and abroad and owns satisfactory reliability and validity [28, 29]. The Cronbach’s alpha coefficient for HHI was 0.928 in the present study.

### Data analyses

SAS University Edition was chosen for data analyses and statistically significance was considered as a two-tailed *P*-value < 0.05. The *χ*^*2*^ test or Cochran-Armitage trend test were used to explore the relationship between independent variables and the prevalence of depressive symptoms. The independent variables whose Chi-square *P*-value was less than 0.05 entered into multivariable logical regression. Potentially confounding demographic variables (gender, grade, monthly disposable income) were not included in the unadjusted model, while they were added to the adjusted model. The Nagelkerke-*R*^*2*^ was the coefficient of determination. The Hosmer-Lemeshow test was used to examine the goodness-of-fit of the models, and *P*-value > 0.05 indicated adequate fitness.

## Results

### Demographic variables and prevalence of depressive symptoms

Of the 1681 college students, the prevalence of depressive symptoms was 56.8% (955). Table 1 displayed the relationship between demographic variables and prevalence of depressive symptoms. 64.8% of the participants were female, who showed a significantly lower prevalence of depressive symptoms than males (*P* < 0.001). Compared with those in grades “1/2”, college students in grades “3/4/5” showed a significantly higher prevalence of depressive symptoms (*P* = 0.010). About 61.7% of the participants had a monthly disposable income between 1001 and 2000 yuan. The results of Cochran-Armitage trend test described a trend that the prevalence of depressive symptoms decreased as the income rose (*P* = 0.010). However, no significant difference in the prevalence of depressive symptoms was found between medical related majors and other majors (*P* = 0.959). 73.8% of college students were from urban areas, and no difference was found in the prevalence of depressive symptoms between urban and rural college students (*P* = 0.163).

**Table 1:** Demographic characteristics of college students

Variables	n (%)	Depressive symptoms		*χ*^*2*^*orZ*	*P*-value
		No, n (%)	Yes, n (%)		
Gender				32.942	< 0.001
Male	592 (35.2)	200 (33.8)	392 (66.2)		
Female	1089 (64.8)	526 (48.3)	563 (51.7)		
Major				0.003	0.959
Others	536 (31.9)	231 (43.1)	305 (56.9)		
Medical related major	1145 (68.1)	495 (43.2)	650 (56.8)		
Grade				6.595	0.010
1/2	1222 (72.7)	551 (45.1)	671 (54.9)		
3/4/5	459 (27.3)	175 (38.1)	284 (61.9)		
Region				1.946	0.163
Rural	441 (26.2)	178 (40.4)	263 (59.6)		
Urban	1240 (73.8)	548 (44.2)	692 (55.8)		
Monthly disposable income				2.582	0.010
≤ 1000	367 (21.8)	147 (40.0)	220 (60.0)		
1001–2000	1038 (61.7)	439 (42.3)	599 (57.7)		
> 2000	276 (16.4)	140 (50.7)	136 (49.3)		

### COVID-19-related perception and prevalence of depressive symptoms

As shown in Table 2, college students with less information sources had a higher prevalence of depressive symptoms than those with more information sources (*P* = 0.009). About 64.2% of college students expressed fear about the COVID-19 pandemic and showed a higher prevalence of depressive symptoms (*P* < 0.001). Nearly 76.2% of the participants thought the recovery of Chinese economy and the health of Chinese citizens would be affected by the global pandemic of COVID-19, and they had a higher prevalence of depressive symptoms (*P* < 0.001). The proportion of college students who thought their social interactions were affected was 37.7%, and these students showed a significantly higher prevalence of depressive symptoms (*P* < 0.001).

**Table 2:** Relationship between COVID-19-related perception and depressive symptoms

Variables	N (%)	Depressive symptoms		*χ*^*2*^	*P*-value
		No, n (%)	Yes, n (%)		
Information sources				6.883	0.009
≤ 3	749 (44.6)	297 (39.7)	452 (60.3)		
≥ 4	932 (55.4)	429 (46.0)	503 (54.0)		
Fear of COVID-19				27.439	< 0.001
No	602 (35.8)	311 (51.7)	291 (48.3)		
Yes	1079 (64.2)	415 (38.5)	664 (61.5)		
Affected by global pandemic				19.558	< 0.001
Moderate	400 (23.8)	211 (52.8)	189 (47.3)		
High	1281 (76.2)	515 (40.2)	766 (59.8)		
Influence on social interaction				13.671	< 0.001
No	1048 (62.3)	489 (46.7)	559 (53.3)		
Yes	633 (37.7)	237 (37.4)	396 (62.6)		

### COVID-19-related behavior and prevalence of depressive symptoms

Table 3 displayed the results of *χ*^*2*^ tests exploring the relationship between COVID-19-related behavior and depressive symptoms. 14.8% of the participants have tried to take some medicine in the hope of preventing COVID-19, and they showed a higher prevalence of depressive symptoms but no statistical difference (*P* = 0.093). College students with sleep problems (47.1%) had a higher prevalence of depressive symptoms than those without sleep problems (*P* < 0.001). Additionally, college students were vulnerable to depressive symptoms when family members (or people living with them) went out for entertainment (*P* < 0.001).

**Table 3:** Relationship between COVID-19-related behavior and depressive symptoms

Variables	N (%)	Depressive symptoms		*χ*^*2*^	*P*-value
		No, n (%)	Yes, n (%)		
Taking preventive medicine				2.826	0.093
No	1433 (85.2)	631 (44.0)	802 (56.0)		
Yes	248 (14.8)	95 (38.3)	153 (61.7)		
Sleep problems				133.323	< 0.001
No	889 (52.9)	501 (56.4)	388 (43.6)		
Yes	792 (47.1)	225 (28.4)	567 (71.6)		
Going Out				22.240	< 0.001
No	1541 (91.7)	692 (44.9)	849 (55.1)		
Yes	140 (8.3)	34 (24.3)	106 (75.7)		

### Perception of online education and prevalence of depressive symptoms

As displayed in Tables 4, 28.9% of the students thought traditional teaching mode was more stressful than online education, and about 30.4% of the students thought online education was more stressful than traditional teaching mode. Moreover, the results of Cochran-Armitage trend test showed there was a significant difference in the prevalence of depressive symptoms among the three groups (*P* < 0.001). 36.3% of the college students were dissatisfied with the online education, and they showed a higher prevalence of depressive symptoms (*P* < 0.001).

**Table 4:** Relationship between perception of online education and depressive symptoms

Variables	N (%)	Depressive symptoms		*χ*^*2*^*orZ*	*P*-value
		No, n (%)	Yes, n (%)		
Perceived more stress				−4.911	< 0.001
Disagree	486 (28.9)	255 (52.5)	231 (47.5)		
Equal	684 (40.7)	282 (41.2)	402 (58.8)		
Agree	511 (30.4)	189 (37.0)	322 (63.0)		
Overall satisfaction				20.720	< 0.001
Satisfaction	1071 (63.7)	507 (47.3)	564 (52.7)		
Dissatisfaction	610 (36.3)	219 (35.9)	391 (64.1)		

### PSS, hope and the prevalence of depressive symptoms

Table 5 showed the differences in PSS, hope between college students with and without depressive symptoms. 71.2% of college students had high level of PSS and showed lower prevalence of depressive symptoms than those with moderate level of PSS (*P* < 0.001). Similarly, nearly 70.6% of the participants had a higher level of hope and they owned a significantly lower prevalence of depressive symptoms than those with moderate level of hope (*P* < 0.001).

**Table 5:** Relationship between PSS, hope and the prevalence of depressive symptoms

Variables	N (%)	Depressive symptoms		*χ*^*2*^	*P*-value
		No, n (%)	Yes, n (%)		
Perceived social support				173.232	< 0.001
Moderate	484 (28.8)	88 (18.2)	396 (81.8)		
High	1197 (71.2)	638 (53.3)	559 (46.7)		
Hope				415.912	< 0.001
Moderate	495 (29.4)	25 (5.1)	470 (94.9)		
High	1186 (70.6)	701 (59.1)	485 (40.9)		

### The results of multivariable logistic regression analysis

Multivariable logistic regression analysis was performed to identify which determinants contributed most to the likelihood of depressive symptoms. The results of the unadjusted model and the model adjusting for potentially confounding demographic factors were reported in Table 6. Gender, grade and monthly disposable income entered the adjusted model as control variables. The Nagelkerke-*R*^*2*^ for the unadjusted and adjusted models were 0.440 and 0.456, respectively. The Hosmer-Lemeshow tests demonstrated the adequate fitness for the unadjusted model (*χ*^*2*^ = 3.813, *P* = 0.874) and adjusted model (*χ*^*2*^ = 8.714, *P* = 0.367).

**Table 6:** Multivariable logistic regression of factors affecting depressive symptoms

Variables	Unadjusted Model	Adjusted Model		
	OR (95% CI)	*P*	OR (95% CI)	*P*
Sleep problems (Yes vs. No)	2.575 (2.022, 3.281)	< 0.001	2.678 (2.094, 3.424)	< 0.001
Going out (Yes vs. No)	1.800 (1.110, 2.918)	0.017	1.775 (1.089, 2.894)	0.021
Perceived more stress 1(Equal vs. Disagree)	1.584 (1.179, 2.127)	0.002	1.621 (1.200, 2.191)	0.002
Perceived more stress 2 (Agree vs. Disagree)	1.665 (1.213, 2.286)	0.002	1.642 (1.191, 2.263)	0.003
Fear of COVID-19 (Yes vs. No)	1.311 (1.021, 1.682)	0.034	1.450 (1.121, 1.876)	0.005
Influence on social interaction (Yes vs. No)	1.377 (1.075, 1.765)	0.011	1.354 (1.053, 1.741)	0.018
Affected by global pandemic(High vs. Moderate)	1.355 (1.022, 1.797)	0.035	1.320 (0.992, 1.757)	0.057
Information source (≥ 4 vs. ≤3)	0.974 (0.765, 1.241)	0.834	0.966 (0.756, 1.234)	0.780
Overall satisfaction (Dissatisfaction vs. Satisfaction)	0.954 (0.733, 1.241)	0.725	0.894 (0.683, 1.170)	0.416
PSS (High vs. Moderate)	0.347 (0.255, 0.473)	< 0.001	0.354 (0.259, 0.484)	< 0.001
Hope (High vs. Moderate)	0.051 (0.033, 0.078)	< 0.001	0.052 (0.034, 0.080)	< 0.001
Gender (Female vs. Male)	–		0.557 (0.427, 0.725)	< 0.001
Grade (3/4/5 vs. 1/2)	–		1.378 (1.046, 1.816)	0.023
Income 1 (1001–2000 vs. ≤1000)	–		1.100 (0.809, 1.495)	0.543
Income 2 (> 2000 vs. ≤1000)	–		0.666 (0.447, 0.993)	0.046
Nagelkerke-*R*^*2*^	0.440	0.456		

Notes: *PSS* perceived social support; income, monthly disposable income; *OR* odds ratio

Sleep problems, family members’ going out, perceived more stress for online education, fear of COVID-19, influence on social interaction and higher grades were considered as risk factors of depressive symptoms. Perceived social support, hope, female and higher monthly disposable income were identified as protective factors against depressive symptoms.

## Discussion

In the present study, the prevalence of depressive symptoms of college students was 56.8%, which was higher than that of the general population (29.1%) [30], and that of Chinese citizens aged ≥18 years old during the COVID-19 pandemic (48.3%) [31]. This suggested that the mental health of college students during the COVID-19 pandemic should not be ignored.

### Risk factors of depressive symptoms

The results of this study suggested the relationship between sleep problems and depressive symptoms. Previous studies have shown that sleep problems are common among college students [32]. As a key indicator of health and happiness, high-quality sleep is good for relieving stress and strengthening the body’s immunity [33]. College students who often suffer from sleep problems are prone to feel tired, lack energy, produce irritability, restlessness and other bad emotions, which may be associated with more serious depressive symptoms [32].

Fear of COVID-19 and family members’ (or people living with them) going out for recreational activities during home quarantine were associated with more severe depressive symptoms in college students. The increasing number of confirmed cases and deaths has contributed to the negative emotions of college students. If there are still family members out, they may be more worried about the health of their family members and themselves, leading to additional depressive symptoms [34]. Moreover, this study found that college students who perceived more stress due to the online education than traditional teaching mode reported a higher prevalence of depressive symptoms. In order not to affect the teaching plan, the universities began to conduct online education. In the face of this new teaching mode, college students with different adaptability may have different degrees of psychological pressure [35, 36].

College students who felt their social interactions were affected had a higher prevalence of depressive symptoms. Normal social interaction is essential in life. Good social relationship lets college students perceive adequate support from classmates, friends and society, which is conducive to physical and mental development [37]. The current pandemic has resulted in social isolation to some extent, and social interactions are affected, which is harmful to the physical and mental health of college students. As Matthews et al. has found, increased loneliness caused by social isolation can be a risk factor for depressive symptoms [38]. Students in higher grades showed a higher prevalence of depressive symptoms. Senior students have heavier learning tasks and also face the pressure of employment. The COVID-19 pandemic brings more inconvenience to senior students, which increases the risk of developing depressive symptoms [39].

### Protective factors of depressive symptoms

Female college students showed a lower prevalence of depressive symptoms. This is consistent with the results of Xu et al. on Chinese college students (31.1% for males vs. 28.4% for females) [21]. However, the gender differences in college students’ psychological problems are still controversial [40], and need to be further studied based on different cultural backgrounds. A higher level of disposable income was possibly a protective factor against depressive symptoms. During the pandemic, higher income gives college students confidence that the supply of food and water can be guaranteed [41].

More importantly, we found that higher level of PSS was associated with lower prevalence of depressive symptoms, which is similar to the findings that social support has a protective effect on college students’ anxiety symptoms during COVID-19 pandemic [15]. As a positive psychological resource, PSS is closely related to an individual’s health and well-being [14]. As a special group, college students’ physical and mental development is not mature, and they are more likely to have psychological problems during the epidemic [30]. The family is an important source of social support, especially during the home quarantine [22]. From the perspective of family support, providing college students with enough care and encouragement will help them to overcome negative psychology and reduce the occurrence of psychological problems [15]. During the current pandemic, the community actively provided assistance to the residents. Free psychological hotline consultations have been put in place nationwide [42]. These are all measures to increase PSS of the residents.

A lower prevalence of depressive symptoms was observed among college students with high level of hope. Visser et al. found that higher levels of hope could moderate the effect of negative life events on depressive symptoms, and this effect did not differ across ethnic groups [18]. Arnau et al. identified the protective effect of hope on college students’ depressive symptoms through two cross-lagged panel models [43]. When facing challenges in life, a college student with hope tends to have a positive attitude, be confident in solving problems, and take a positive coping style [44]. As COVID-19 continues to spread globally, psychological problems have become widespread, not just among patients and medical staff [45]. Due to the unavailability of vaccines, the unpredictability of the pandemic and the indefinite quarantine, the Chinese people are facing huge psychological pressure, and the college students are no exception [30]. Strategies and measures to improve college students’ level of hope may help to reduce the depressive symptoms.

### Strengths and limitations

COVID-19 spreads rapidly all over the world. To some extent, this study adds to the research on college students’ mental health and public health. We studied the relationship between demographic characteristics, COVID-19-related perception and behavior, perception of online education, positive psychological factors and depressive symptoms among college students at the same time.

Some limitations should be discussed. First, this cross-sectional study cannot allow us to demonstrate the causal relationship between variables. Second, self-reported questionnaire was conducted online, which will lead to recall bias and response bias to some extent. We have tried to reduce these biases by using CES-D, MSPSS and HHI whose satisfactory reliability and validity have been verified in Chinese. Finally, in addition to the variables we took into account, there may be other factors that were associated with the prevalence of depressive symptoms in college students.

## Conclusions

Chinese college students have experienced a higher prevalence of depressive symptoms during the COVID-19 pandemic. It is important to find ways to alleviate the pressure and fear of college students, to provide them with more social support, and to help them adapt to the changes in learning style and lifestyle. The government can open free psychological hotline consultations to help college students solve their psychological problems. The media should release correct information timely and prevent the spread of rumors. Universities can actively organize health education activities and encourage college students to arrange their time reasonably and take the initiative to find a suitable way to relieve stress during home quarantine.

## Supplementary Information

**Additional file 1.**

## Data Availability

The data that support the findings of this study are available on request from the corresponding author.

## Abbreviations

CES-D: Center for Epidemiologic Studies Depression scale
MSPSS: Multi-Dimensional Scale of Perceived Social Support
HHI: Herth Hope Index
WHO: World Health Organization
PSS: Perceived social support
OR: Odds ratio

## References

[1] WHO (2020). Coronavirus disease (COVID-19) weekly epidemiological update.

[2] Ahmad A, Mueller C, Tsamakis K (2020). Covid-19 pandemic: a public and global mental health opportunity for social transformation?. BMJ.

[3] Li Z, Ge J, Yang M, Feng J, Liu C, Yang C (2020). Vicarious traumatization: a psychological problem that cannot be ignored during the COVID-19 pandemic. Brain Behav Immun.

[4] Auerbach RP, Mortier P, Bruffaerts R, Alonso J, Benjet C, Cuijpers P (2018). WHO world mental health surveys international college student project: prevalence and distribution of mental disorders. J Abnorm Psychol.

[5] Lei X, Xiao L, Liu Y, Li Y (2016). Prevalence of depression among Chinese University students: a meta-analysis. PLoS One.

[6] Blanco C, Okuda M, Wright C, Hasin DS, Grant BF (2008). Mental health of college students and their non-college-attending peers: results from the National Epidemiologic Study on alcohol and related conditions. Arch Gen Psychiatry.

[7] Sarokhani D, Delpisheh A, Veisani Y, Sarokhani MT, Manesh RE, Sayehmiri K (2013). Prevalence of Depression among University Students: A Systematic Review and Meta-Analysis Study. Depress Res Treat.

[8] Acharya L, Jin L, Collins W (2018). College life is stressful today - emerging stressors and depressive symptoms in college students. J Am Coll Heal.

[9] Verboom CE, Sijtsema JJ, Verhulst FC, Penninx BWJH, Ormel J (2014). Longitudinal associations between depressive problems, academic performance, and social functioning in adolescent boys and girls. Dev Psychol.

[10] Liu Y, Zhang N, Bao G, Huang Y, Ji B, Wu Y (2019). Predictors of depressive symptoms in college students: a systematic review and meta-analysis of cohort studies. J Affect Disord.

[11] Ullah R, Amin S (2020). The psychological impact of COVID-19 on medical students [letter]. Psychiatry Res.

[12] Seligman MEP, Csikszentmihalyi M (2000). Positive psychology: an introduction. Am Psychol.

[13] Arden MA, Chilcot J (2020). Health psychology and the coronavirus (COVID-19) global pandemic: a call for research. Br J Health Psychol.

[14] Yang C, Xia M, Han M, Liang Y (2018). Social support and resilience as mediators between stress and life satisfaction among people with substance use disorder in China. Front Psychiatry.

[15] Cao W, Fang Z, Hou G, Han M, Xu X, Dong J (2020). The psychological impact of the COVID-19 epidemic on college students in China. Psychiatry Res.

[16] Rankin JA, Paisley CA, Mulla MM, Tomeny TS (2018). Unmet social support needs among college students: relations between social support discrepancy and depressive and anxiety symptoms. J Couns Psychol.

[17] Poorgholami F, Abdollahifard S, Zamani M, Jahromi MK, Jahromi ZB (2015). The effect of stress management training on Hope in hemodialysis patients. Glob J Health Sci.

[18] Visser PL, Loess P, Jeglic EL, Hirsch JK (2013). Hope as a moderator of negative life events and depressive symptoms in a diverse sample. Stress Health.

[19] Radloff LS (1977). The CES-D scale: a self-report depression scale for research in the general population. Appl Psychol Meas.

[20] Tao S, Wu X, Zhang Y, Zhang S, Tong S, Tao F (2017). Effects of sleep quality on the association between problematic Mobile phone use and mental health symptoms in Chinese college students. Int J Environ Res Public Health.

[21] Xu Y, Qi J, Yang Y, Wen X (2016). The contribution of lifestyle factors to depressive symptoms: a cross-sectional study in Chinese college students. Psychiatry Res.

[22] Zimet GD, Dahlem NW, Zimet SG, Farley GK (1988). The multidimensional scale of perceived social support. J Pers Assess.

[23] Li L, Zhao Y, Li H (2020). Assessment of anxiety and depression in patients with incidental pulmonary nodules and analysis of its related impact factors. Thorac Cancer.

[24] Zhang Y, Jin S (2016). The impact of social support on postpartum depression: the mediator role of self-efficacy. J Health Psychol.

[25] Zhong Y, Wang J, Nicholas S (2020). Social support and depressive symptoms among family caregivers of older people with disabilities in four provinces of urban China: the mediating role of caregiver burden. BMC Geriatr.

[26] Herth K (1991). Development and refinement of an instrument to measure hope. Sch Inq Nurs Pract.

[27] Li L, Lin M, Liang J, Hu Q, Chen D, Lan M (2017). Effects of intrinsic and extrinsic factors on the level of Hope and psychological health status of patients with cervical Cancer during radiotherapy. Med Sci Monit.

[28] Gao Y, Yuan L, Pan B, Wang L (2019). Resilience and associated factors among Chinese patients diagnosed with oral cancer. BMC Cancer.

[29] Wang Q, Xu W, Ren L, Wang W, Wang Y (2019). The relationship between hope and post-traumatic stress disorder in Chinese shidu parents: the mediating role of perceived stress. J Affect Disord.

[30] Huang Y, Zhao N (2021). Mental health burden for the public affected by the COVID-19 outbreak in China: who will be the high-risk group?. Psychol Health Med..

[31] Gao J, Zheng P, Jia Y, Chen H, Mao Y, Chen S (2020). Mental health problems and social media exposure during COVID-19 outbreak. PLoS One.

[32] Williams AB, Dzierzewski JM, Griffin SC, Lind MJ, Dick D, Rybarczyk BD (2020). Insomnia disorder and behaviorally induced insufficient sleep syndrome: prevalence and relationship to depression in college students. Behav Sleep Med.

[33] Lange T, Dimitrov S, Born J (2010). Effects of sleep and circadian rhythm on the human immune system. Ann N Y Acad Sci.

[34] Li S, Wang Y, Xue J, Zhao N, Zhu T (2020). The impact of COVID-19 epidemic declaration on psychological consequences: a study on active Weibo users. Int J Environ Res Public Health.

[35] Chow SKY, Choi EKY (2019). Assessing the mental health, physical activity levels, and resilience of Today’s Junior college students in self-financing institutions. Int J Environ Res Public Health.

[36] Zong J, Cao X, Cao Y, Shi Y, Wang Y, Yan C (2010). Coping flexibility in college students with depressive symptoms. Health Qual Life Outcomes.

[37] Umberson D, Montez JK (2010). Social relationships and health: a flashpoint for health policy. J Health Soc Behav.

[38] Matthews T, Danese A, Wertz J, Odgers C, Ambler A, Moffitt T (2016). Social isolation, loneliness and depression in young adulthood: a behavioural genetic analysis. Soc Psychiatry Psychiatr Epidemiol.

[39] Huebschmann NA, Sheets ES (2020). The right mindset: stress mindset moderates the association between perceived stress and depressive symptoms. Anxiety Stress Coping.

[40] de Sá Junior AR, Liebel G, de Andrade AG, Andrade LH, Gorenstein C, Wang YP (2019). Can gender and age impact on response pattern of depressive symptoms among college students? A Differential Item Functioning Analysis. Front Psychiatry.

[41] Hodgkinson S, Godoy L, Beers LS, Lewin A (2017). Improving mental health access for low-income children and families in the primary care setting. Pediatrics..

[42] CCTV. http://news.cctv.com/2020/02/14/ARTI3ZhIqxhLAD0jkmmViNC5200214.shtml. Accessed 14 February 2020. [News in Chinese].

[43] Arnau RC, Rosen DH, Finch JF, Rhudy JL, Fortunato VJ (2007). Longitudinal effects of hope on depression and anxiety: a latent variable analysis. J Pers.

[44] Snyder CR, Harris C, Anderson JR, Holleran SA, Irving LM, Sigmon ST (1991). The will and the ways: development and validation of an individual-differences measure of hope. J Pers Soc Psychol.

[45] Xiang YT, Yang Y, Li W, Zhang L, Zhang Q, Cheung T (2020). Timely mental health care for the 2019 novel coronavirus outbreak is urgently needed. Lancet Psychiatry.

