# Supplementary material for: Prevalence and its associated factors of depressive symptoms among Chinese college students during the COVID-19 pandemic
Source: BMC Psychiatry. 2021 Jan 29;21:66. doi: 10.1186/s12888-021-03066-9 (PMC7845579; doi:10.1186/s12888-021-03066-9)
Supplement: Supplementary file 1 — Additional file 1. [file 12888_2021_3066_MOESM1_ESM.docx]

**Prevalence and its associated factors of depressive symptoms among Chinese college students during the COVID-19 pandemic**

**Mingli Yu^1^, Fangqiong Tian^1^, Qi Cui^1^, Hui Wu^1*^**

^1^Department of Social Medicine, School of Public Health, China Medical University, 110122, China

*** Correspondence:** Hui Wu, **[hwu@cmu.edu.cn](mailto:hwu@cmu.edu.cn)**

**The list of self-designed items**

**COVID-19-Related Perception**

1. Information sources was evaluated by the question “What are the main channels for you to know about the harm and epidemic situation of COVID-19?” with eight possible options: the people around, WeChat, Weibo, Media website or mobile clients, TV, newspaper, radio or Government announcements. According to the number of information sources, the answers were further converted to “≤3” and “≥4”.

2. Fear of COVID-19 was measured by this question “Are you terrified by the prevalence of COVID-19 and its possible harm to health, economy, etc.?” with two answers: “no” and “yes”.

3. Affected by global pandemic was evaluated by the question “To what extent do you think the global pandemic of COVID-19 will affect the recovery of Chinese economy and the health of Chinese citizens?” and the responses were categorized into “moderate” (mild influence/moderate influence) and “high” (great influence).

4. Influence on social interaction was measured by the following question “Were your social interactions with friends or classmates affected during the COVID-19 pandemic?” with the answers being divided into “no” (never/rarely) and “yes” (sometimes/often).

**COVID-19-Related Behavior**

1. Taking preventive medicine was assessed by this question “Have you ever taken any medicine in the hope of preventing COVID-19?” with two options: “yes” and “no”.

2. “Did you suffer from sleep problems at night during home quarantine?” was used to evaluate sleep problems. The responses were further divided into “no” (never) and “yes” (sometimes/often).

3. Going out was evaluated by this question “Since the outbreak of COVID-19, has anyone in your family (or living you together) still been out for entertainment, such as chatting, having parties and so on?” with two options: “no” and “yes”.

**Perception of Online Education**

1. Perceived more stress was measured by the question “Do you find online education more stressful than traditional teaching mode?” with three options: “disagree”, “equal” and “agree”.

2. Overall satisfaction with online education was divided into “satisfaction” and “dissatisfaction”.
